# Combining Gene Mutation with Expression of Candidate Genes to Improve Diagnosis of Escobar Syndrome

**DOI:** 10.3390/genes13101748

**Published:** 2022-09-27

**Authors:** Dorra Najjar, Asma Chikhaoui, Sinda Zarrouk, Saifeddine Azouz, Wafa Kamoun, Nabil Nassib, Sami Bouchoucha, Houda Yacoub-Youssef

**Affiliations:** 1 Laboratory of Biomedical Genomics and Oncogenetics (LR16IPT05), Institut Pasteur de Tunis, Université Tunis El Manar, Tunis 1002, Tunisia; 2 Genomics Platform, Institut Pasteur de Tunis (IPT), Tunis-Belvédère, Tunis 1002, Tunisia; 3 Service Orthopédie Pédiatrique, Hôpital d’Enfant Béchir Hamza, Tunis 1000, Tunisia

**Keywords:** Escobar syndrome, rare neuromuscular disease, WES, nemaline myopathy, *TPM2*, *CHRNG*, genetic modifiers, rt-qPCR, *POLG1*, *IGF-1*

## Abstract

Escobar syndrome is a rare, autosomal recessive disorder that affects the musculoskeletal system and the skin. Mutations in the *CHRNG* and *TPM2* genes are associated with this pathology. In this study, we conducted a clinical and genetic investigation of five patients and also explored via in silico and gene expression analysis their phenotypic variability. In detail, we identified a patient with a novel composite heterozygous variant of the *CHRNG* gene and two recurrent mutations in both *CHRNG* and *TPM2* in the rest of the patients. As for the clinical particularities, we reported a list of modifier genes in a patient suffering from myopathy. Moreover, we identified decreased expression of *IGF*-1, which could be related to the short stature of Escobar patients, and increased expression of *POLG1* specific to patients with *TPM2* mutation. Through this study, we identified the genetic spectrum of Escobar syndrome in the Tunisian population, which will allow setting up genetic counseling and prenatal diagnosis for families at risk. In addition, we highlighted relevant biomarkers that could differentiate between patients with different genetic defects.

## 1. Introduction

Multiple pterygium syndrome (MPS) is a rare genetic disorder belonging to the group of arthrogryposis diseases, and it is distinguished by the presence of pterygia. This syndrome is due to fetal akinesia [1]. MPS includes two major forms, namely, acute and mild. The acute form is lethal multiple pterygium syndrome LMPS (OMIM: 253290); it leads to the death of the fetus shortly after birth. The mild form is nonlethal MPS, commonly known as “Escobar syndrome” (OMIM: 265000), referring to Victor Escobar, who classified this syndrome [2].

Genetic studies have shown that Escobar syndrome is an autosomal recessive disorder, linked in most cases to mutations in the *CHRNG* gene [3,4]. This gene encodes the γ-subunit of the acetylcholine receptor (AChR) of skeletal muscles during fetal life (a fetal form of the receptor) or denervated cells [4]. The AChR is composed of five subunits (2α1, β, δ, and γ/ε) [5]. Mutations in *CHRNG* lead to phenotypes, including congenital multiplex arthrogryposis, multiple pterygium, facial dysmorphism, scoliosis, and prenatal growth failure. Mutations in the γ-subunit are the major cause of MPS in its two forms: Escobar syndrome and LMPS. Meanwhile, there is no correlation between phenotype and genotype, which means that the same mutation can lead to either the lethal or nonlethal form of MPS [6,7].

The γ-subunit is expressed in fetal muscle during motor development on the surface of the sarcolemma, contributing to neuromuscular junction (NMJ) development [8]. Beginning at the 33rd week of pregnancy, there is a progressive switch of the fetal receptor form (2α1βδγ) to the adult form (2α1βδε) of the AChR. It consists of the switch of the γ-subunit to the ε-subunit [9,10]. For this, there is no longer any risk of disease progression after birth [3]. However, partial inactivation of the NMJ in the fetus causes fetal akinesia, which leads to muscle weakness, severe arthrogryposis, and pterygia [11]. In addition, some Escobar patients present a nonspecific myopathy revealed by electromyography (EMG) and whole-body magnetic resonance imaging (WBMRI) techniques [11,12].

In the literature, 75 Escobar patients have been described, harboring 26 mutations in the *CHRNG* gene [11,13,14]. In Tunisia, only one patient has been clinically diagnosed with Escobar syndrome so far [15]. Thus, a genetic investigation is recommended to set up genetic counseling and prenatal diagnosis for families at risk and early follow-up of patients.

Other genes are related to MPS. Indeed, mutations in *CHRNA1*, *CHRND* [4,16], and *TPM2* have been described to be associated with Escobar syndrome [17,18,19,20]. This genetic heterogeneity makes genetic investigation challenging.

Whole-exome sequencing (WES) is considered a highly sensitive technique that facilitates the diagnosis of rare and complex genetic diseases. It allows early diagnosis of fetal akinesia that is not detected by conventional ultrasound [21,22]. In addition, with genetic and clinical spectrum heterogeneity, it is important to identify biomarkers that are implicated in the physiopathology of this disease to improve Escobar syndrome diagnosis.

Interestingly, more than 55% of Escobar patients present short stature. In most studies, this short stature has been associated with a deficiency in insulin growth factor-1 (*IGF-1*), which contributes to the skeletal development of the fetus [23]. Acetylcholine (Ach) deficiency in mice leads to a decrease in *IGF-1* and is considered a modulator hormone for the secretion of *IGF-1* [24]. Furthermore, *IGF-1* promotes the development, growth, differentiation, and maintenance of the strength of bones and muscles [25]. This makes *IGF-1* an interesting biomarker in investigating Escobar syndrome.

Some cases of patients with *TPM2* mutations presented with nemaline myopathy (NM) associated with the Escobar syndrome phenotype [17,18,19]. Previous studies have shown that patients with NM have subsarcolemmal accumulations of abnormal mitochondria and nemaline rods [26,27,28]. *POLG1* encodes a DNA polymerase involved in mitochondrial DNA (mtDNA) replication. Its alteration is associated with many types of myopathies such as NM, and it is considered a biomarker for mitochondrial diseases [29].

In this study, we used WES and Sanger sequencing to identify causative variants in the largest cohort of Escobar syndrome in the Tunisian population. In addition, we highlight relevant biomarkers *IGF-1* and *POLG1* that could improve and guide Escobar diagnosis.

## 2. Materials and Methods

### 2.1. Clinical Data Collection

This study was carried out in accordance with the Declaration of Helsinki and approved by Institute Pasteur Ethics Committee in Tunisia under Ethical Accordance Number 2021/10/E/V1. After obtaining written informed consent from patients and their tutors (for minors), blood samples were obtained from the pediatric orthopedic department (Hospital Bechir Hamza, Tunis).

Five patients were enrolled in the study. These patients underwent routine general examinations since birth. Clinical and genealogical data were collected under the supervision of the referral doctor. X-ray examination was performed for all patients suspected of Escobar syndrome, and electromyography was performed for three patients (Patients 1, 2, and 4) who were available at the time of the study.

### 2.2. DNA Extraction

Blood samples were collected from probands and their parents. DNA extraction was performed using the DNeasy Blood & Tissue Kit (Qiagen, Hilden, Germany), according to the manufacturer’s instructions. DNA quality was assessed using the DENOVIX DS-11 nanodrop spectrophotometer.

### 2.3. Sanger Sequencing

Molecular investigation of the full coding regions of the *CHRNG* gene and confirmation of the variants detected following WES data analysis was performed using the Sanger sequencing technique with the ABI Prism 3500 sequencer (Applied Biosystems, Foster City, CA, USA).

### 2.4. Whole Exome Sequencing and Bioinformatics Analysis

Two patients (Patients 2 and 3) underwent whole-exome sequencing (WES). Libraries were pooled together, and reads were sequenced on the Illumina NovaSeq 6000 platform (Illumina, San Diego, CA, USA). Sequence quality control was performed with FastQC (https://www.bioinformatics.babraham.ac.uk/projects/fastqc/) (accessed on 1 April 2020). Read mapping to Genome Reference Consortium Human Build 38 (GRCh38) was performed with Bowtie2 [30] with default parameters. Filtration, trimming, removing errors, and low-quality reads (Q20) of Fastq data were performed using the TrimGalore and Picard tools (MarkDuplicates) (https://picard.sourceforge.net) (accessed on 9 April 2020). SNP and INDEL calling, together with advanced variant annotation, were performed using GATK4, and annotation was performed with ANNOVAR. WES revealed an average of 38,064 homozygous variants and an average of 66,238 heterozygous variants within the capture regions on average for each patient. Variants were further filtered using VarAFT according to the population frequency of variants that was determined using databases such as GenomAD, 1000 Genomes Project, and Exome Aggregation Consortium (ExAC) v0.3. Only novel and rare variants were included in these analyses, defining rare as minor allele frequency (MAF) < 0.5%. They were subsequently filtered based on their type and genomic localization and in silico pathogenicity prediction according to UMD Predictor (http://umd-predictor.eu/) (accessed on 16 April 2021), SIFT (http://sift.jcvi.org/) (accessed on 16 April 2021), PolyPhen-2 (http://genetics.bwh.harvard.edu/pph2/) (accessed on 16 April 2021), MutationTaster (http://www.mutationtaster.org/) (accessed on 16 April 2021), and ClinVar (https://www.st-va.ncbi.nlm.nih.gov/clinvar/) (accessed on 16 April 2021). Online software such as UniProt (https://www.uniprot.org/) (accessed on 16 April 2021), PROVEAN (http://provean.jcvi.org/seq_submit.php) (accessed on 16 April 2021), M-Cap (http://bejerano.stanford.edu/mcap (accessed on 16 April 2021)), MÜPRO (http://mupro.proteomics.ics.uci.edu/) (accessed on 16 April 2021), and I-Mutant (https://folding.biofold.org/i-mutant//i-mutant2.0.html) (accessed on 16 April 2021) was used to analyze the protein structure and predict the conserved and functional domains. Other prediction tools were used, such as Human Splicing Finder (www.umd.be/HSF/) (accessed on 16 April 2021), varSEAK (https://varseak.bio/index.php) (accessed on 16 April 2021), and MaxEntScan (http://hollywood.mit.edu/burgelab/maxent/Xmaxentscan_scoreseq.html) (accessed on 16 April 2021), to predict the effect of splice site variants. Pathogenicity assessment was performed according to the American College of Medical Genetics (ACMG) guidelines [31], using all prediction tools that were confirmed by the Sanger sequencing technique. For the analysis of oligogenic inheritance between different variants, whose interaction could be the cause of oligogenic diseases, we carried out digenic combinations using the bioinformatics platform Orval (https://orval.ibsquare.be/) (accessed on 30 September 2021) [32].

### 2.5. RNA Extraction and Real-Time Quantitative PCR

Total RNA was isolated from peripheral blood mononuclear cells (PBMCs) from all patients and from three healthy age-matched donors (the mean age was 14, ranging from 9 to 22 years old) using TRIzol reagent (No.: BCBL7327V, Sigma Aldrich, Darmstadt, Germany). cDNA was obtained by reverse transcription using SuperScript II, according to the manufacturer’s instructions, then treated with DNase I, Amplification Grade (No.: 18068015, Invitrogen, Carlsbad, CA, USA). We tested the expression of the *IGF-1* and *POLG1* genes using the SYBR Green-Based qPCR technique. Primers were selected from the PrimerBank database (https://pga.mgh.harvard.edu/primerbank/) (accessed on 14 April 2021) and articles. Manual constructions were performed for the primers of candidate genes (Table 1). The LightCycler 480 SYBR Green I Master Mix (No: 04 707 516 001, Roche, Darmstadt, Germany) was used, according to the manufacturer’s instructions. Q-PCR was performed on the LightCycler 480 System (Roche Diagnostics). Relative quantification Ct values were obtained from the threshold cycle number of a duplicate test and normalized to the healthy samples. *PPIA* and *RLP0* were used as housekeeping genes.

### 2.6. Statistical Analysis

Differences in the fold-change expression of *IGF-1* and *POLG1* between Escobar patients and healthy donors following qPCR were assessed by one-way ANOVA. The level of significance was set at 0.05 (*p* < 0.05) with a 95% confidence interval. Analyses were performed using GraphPad software version 9.00 for Windows (GraphPad Software, La Jolla, CA, USA, www.graphpad.com, accessed on 1 July 2020).

## 3. Results

### 3.1. Clinical Investigation

Clinical follow-up was set up for the five patients with clinical suspicion of Escobar syndrome.

Detailed clinical characterization is summarized in Table 2.

#### 3.1.1. Prenatal Abnormalities

The standard inquiry performed with the families revealed that for two patients (Patients 3 and 4), their mothers reported hypokinesia of the fetus, and that for the mother of Patient 3, she complained of the abnormal movements of the fetus. These patients were born through cesarean section delivery. For Patient 2, during pregnancy, the mother suffered from chronic fever.

All patients presented the same major clinical features with differences in the severity of each criterion.

#### 3.1.2. Facial and Skin Anomalies

**Figure 1:**
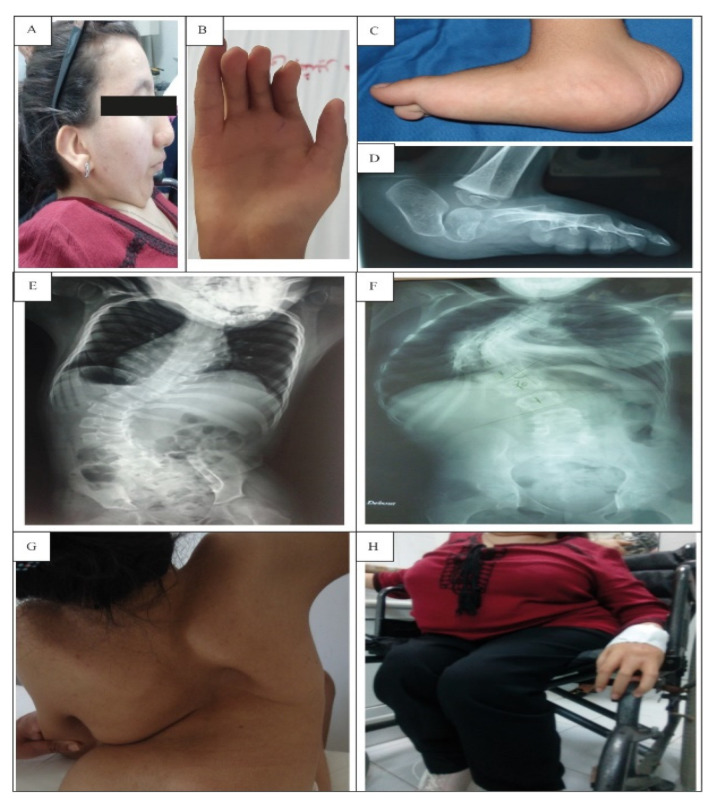
Radiological imaging and photos of patients with Escobar syndrome: (**A**) facial dysmorphism in Escobar patients; (**B**) camptodactyly; (**C**,**D**) foot deformities of Patient 1; (**E**–**G**) scoliosis of Patients 1–3, respectively; (**H**) paraplegia of Patient 3.

Clinical examination indicated facial dysmorphism (Figure 1A), which was manifested by low-set ears, mild ptosis, down-slanted palpebral fissures, a small mouth, and a low nose bridge. In addition, these patients present a short neck and multiple pterygium (in the neck and articulations).

#### 3.1.3. Skeletal Deformities

All patients who arrived at our department for examination presented multiple congenital arthrogryposis covered by multiple pterygia (Figure 1B) and short stature. In addition, they displayed psychomotor delay. Walking without support was acquired in three cases (Patients 1, 4, and 5) at the age of 2. Patient 2 was unable to walk alone and needed support. As for Patient 3, she had motricity delay with an abnormal walking ability at 3 years old due to severe flexion contractures of the knees. At the age of 4, she became unable to walk and needed a wheelchair (Figure 1H) (Table 2). These two patients (Patients 2 and 3) presented reduced muscle bulk and fatigability in the lower limbs, especially for Patient 3, who presented severe muscle weakness and bone deformities. Electromyography (EMG) was performed for Patients 1, 2, and 4. Normal results were obtained but were situated in the lower acceptable range. These patients refused myogenic EMG trace to confirm the myopathy (Table 2). Scoliosis was observed following X-ray examination in four patients (Patients 1, 2, 3, and 5) (Figure 1E–G). Bilateral vertical talus was observed in three patients (Patients 1, 3, and 4) (Figure 1C,D). Dislocation of the hips was observed in Patient 5.

#### 3.1.4. Genealogical Data of the Patients

**Figure 2:**
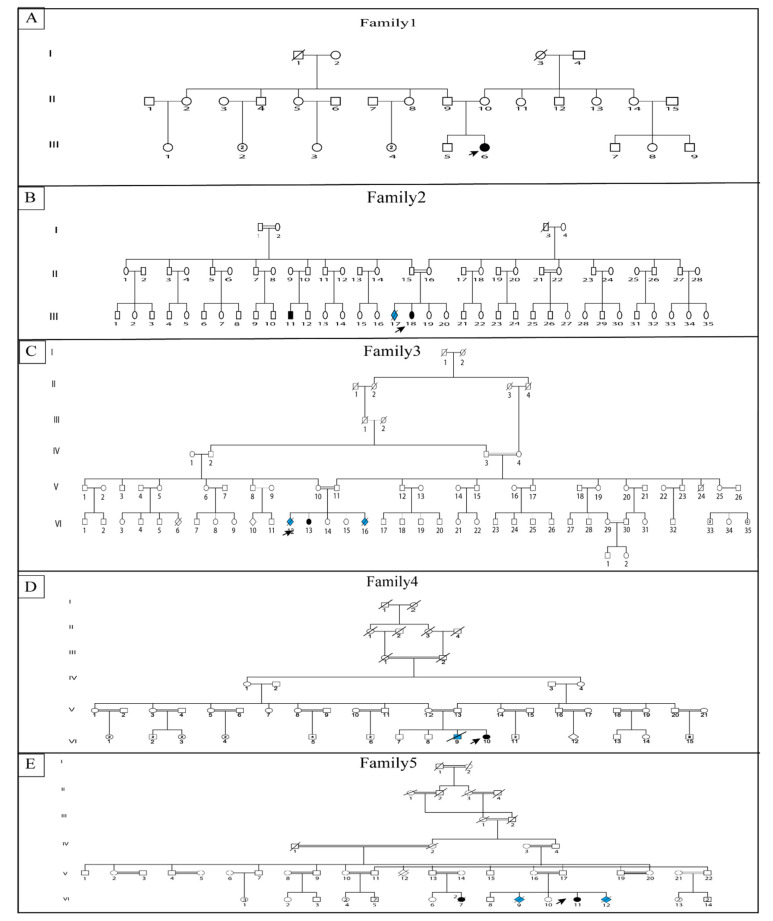
Pedigree describing affected Escobar family members (**A**–**E**) describe the five studied families. Filled symbols represent affected individual; arrows represent proband; blue filled symbols represent possible diseased individuals related to LMPS open symbols represent unaffected individuals, each generation is labelled I, II from the oldest to the most recent.

This cohort included five females from six unrelated families (Families 1–5). Consanguinity, examined during genealogical data inquiry (Figure 2), was found in three families (Families 3–5), while endogamy was reported for the two other families. All patients were from North Tunisia, except Families 4 and 5, who were from South Tunisia.

#### 3.1.5. History of LMPS Cases

A history of LMPS cases was noted for four families (Families 2–5). Indeed, several spontaneous abortions during previous pregnancies for three families (Families 2, 3, and 5) were reported. For Patient 4, her sibling was born with multiple pterygia and joint contractures and died at the age of 6 months from respiratory failure and growth retardation. The clinical manifestations were consistent with the diagnosis of LMPS.

### 3.2. Genetic Findings

In order to determine the genetic causes of Escobar syndrome in these patients, we first performed Sanger sequencing for mutations associated with the *CHRNG* gene (NM_005199.5). Secondly, WES was performed for two unresolved cases.

#### 3.2.1. Patient 4 and 5

**Figure 3:**
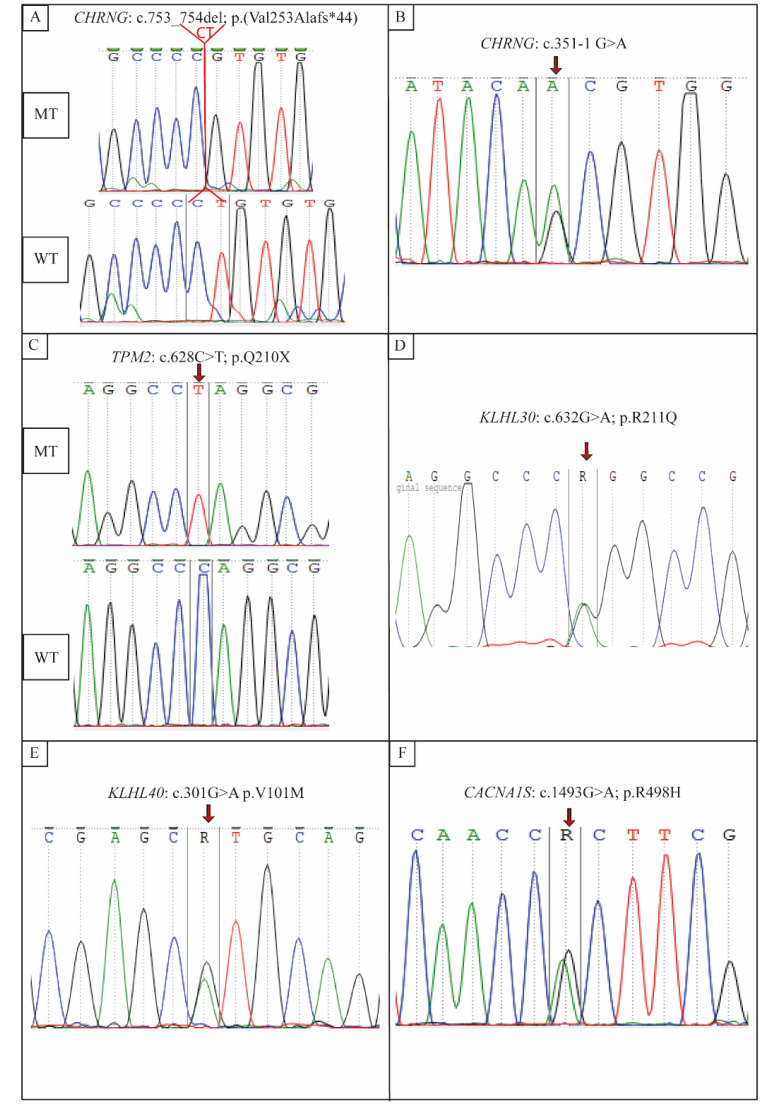
Electropherograms showing genetic particularities in Tunisian Escobar patients: (**A**) mutation in *CHRNG* gene, at a homozygous state in Patients 4 and 5; (**B**) mutation in *CHRNG* gene, at a heterozygous state in Patient 1 and her mother; (**C**) mutation in *TPM2* gene, at a homozygous state in Patients 2 and 3; (**D**) mutation in *KLHL30* gene, at a heterozygous state in Patients 2 and 3; (**E**) mutation in *KLHL40* gene, at a homozygous state in Patients 2 and 3; (**F**) mutation in *CACNA1S* gene, at a heterozygous state in Patient 3.

Both patients (Patients 4 and 5) harbored homozygous deletion in the *CHRNG* gene (NM_005199.5) located in exon 7 c.753_754del/rs767503038 (Figure 3A). This frameshift variant leads to a premature stop codon (p.Val253Alafs*44) and was described as pathogenic according to ClinVar.

#### 3.2.2. Patient 1

**Figure 4:**
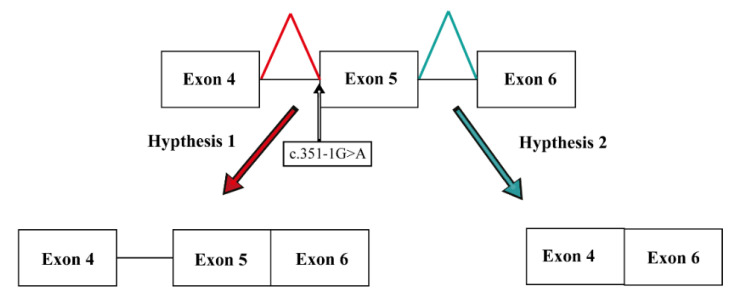
Schematic presentation of possible effect of c.351-1 G>A *CHRNG* splice site mutation. Hypothesis 1: retention of introns 4–5; Hypothesis 2: exon skipping.

The sequencing of exons of the *CHRNG* gene revealed a novel heterozygous mutation in the splice site of exon 5 detected in Patient 1 and her mother. It is a novel variant (c.351-1G>A) located in the 3′ extremities of the acceptor splice site of exon 5 (rs: 761413806) (Figure 3B and Figure 4, Table 3).

#### 3.2.3. Patients 2 and 3

For these two patients, Sanger sequencing of all exons of the *CHRNG* gene did not reveal any pathogenic variant. Therefore, samples underwent WES. Different bioinformatic pipelines were used. We filtered and ranked the best candidate genes and identified several rare variants. We identified a nonsense mutation in the two patients in a homozygous state located in the *TPM2* gene (NM_003289.4) in exon 6 (c.628C>T; p.Q210*/rs199476154) (Figure 3C), which was further validated via Sanger sequencing and confirmed in the parents for segregation.

### 3.3. Genetic Particularity of Patient 3

In order to explain the clinical heterogeneity between the two patients with *TPM2* mutation, and especially for Patient 3, who was paralyzed at a young age, we investigated variants in other genes involved in nemaline myopathy (NM), akinesia/hypokinesia, arthrogryposis, and LMPS.

We identified three novel variants in three different genes solely in Patient 3 among all the other patients. All variants were confirmed by Sanger sequencing in all available family members, as well as for its segregation in the parents.

The first variant was a de novo mutation detected in the *KLHL30* (NM_198582.4) gene in exon 2 c.632G>A; p.R211Q. This variant was in a heterozygous state (Figure 3D).

The second variant was detected in the *KLHL40* gene (NM_152393) located in exon 1 c.301G>A p.V101M in a heterozygous state with a parental inheritance (Figure 3C).

The third variant was a heterozygous nonsense mutation located in the *CACNA1S* gene (NM_000069.3) in exon 11, c.1493G>A; p.R498H (Figure 3F). It was inherited from her mother.

Online prediction tools such as MutationTaster, SIFT, POLYPhen 2, LRT, Mutation Assessor, ProVean, M-Cap, MÜPRO, and I-Mutant showed that these variants are predicted to be pathogenic due to muscle weakness.

All the variants identified in this work were not listed in the ExAC exome database, nor in the 1000 Genome database, HGMD, and GnomAD.

#### Oligogenic Inheritance

As a part of our approach to identifying if the novel detected variants that are not related to Escobar syndrome impact the phenotype in some patients as genetic modifiers, we analyzed the interaction of the oligogenic genes using the Orval prediction tool. A high score of pathogenicity interaction in three pathogenic variant pairs (including the variant in the *TPM2* gene) causing the disease was found (Table 4).

### 3.4. Decreased IGF1 Expression in Escobar Patients with Short Stature

**Figure 5:**
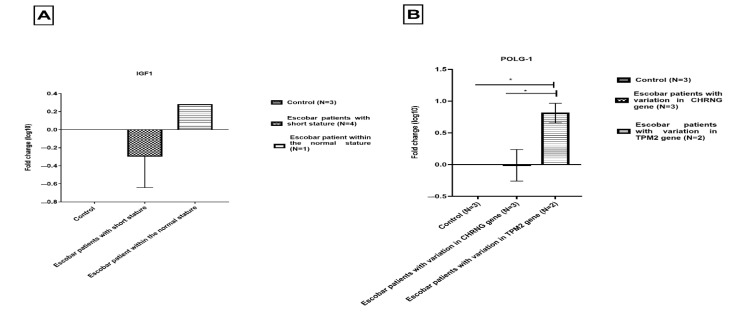
*IGF-1* and *POLG1* genes’ expression in Escobar patients (**A**) *IGF1* expression in Escobar patients of short and normal stature (**B**) *POLG1* in Escobar patients with and without *TPM2* variant. Data are presented as Log10 fold change. Results are presented as mean ± standard error for qPCR normalized to 2 housekeeping genes RPLP0 and PPIA (* *p* < 0.5).

We further investigated the expression of *IGF-1,* which could be associated with the short stature of Escobar patients. Low *IGF-1* expression was found in four patients presenting short stature compared to healthy donors (fold change (Log10) = −0.29 ± 0.3). Normal expression of this gene was found for Patient 4, who presented a height within the normal range (Figure 5).

### 3.5. Alteration of POLG1 Expression in Escobar Patients and Its Possible Link with TPM2 Mutation

We investigated the expression of *POLG1* to explain the reduced muscle bulk and to confirm the differential diagnosis of mitochondrial myopathy as secondary pathology related to NM in some patients. For Patients 2 and 3, we identified a significant increase in *POLG1* expression compared to healthy donors (fold change (Log10) = 0.8 ± 0.15; *p* = 0.016), while normal expression was found in patients with mutations in the *CHRNG* gene (fold change (Log10) = −0.01 ± 0.2) (Figure 5).

## 4. Discussion

### 4.1. Clinical and Instrumental Findings

Arthrogryposis is a clinical phenotype characterized by a contracture of two or more joints of the body [33]. It is the consequence of fetal akinesia, which leads to the formation of connective tissue around the joint responsible for joint stiffness [34]. This highlights the importance of intrauterine movement for normal osteoarticular development of the fetus [35,36]. Escobar syndrome is a particular form of arthrogryposis. It is a rare genetic disease whose diagnosis is based on the presence of clinical symptoms affecting mainly the musculoskeletal system [11]. This syndrome is the nonlethal form of multiple pterygium syndrome.

In this work, we report five patients with a clinical diagnosis of Escobar syndrome who were genetically and clinically investigated. All patients had facial dysmorphism such as ptosis, down-palpebral slanting, a low nose bridge, a small mouth, and low-set ears, which characterize 91% of Escobar patients [11]. Four out of five patients had short stature, except for Patient 4, who presented a height within the normal range. In fact, short stature characterizes more than 55% of Escobar patients [11]. Other characteristics of Escobar syndrome are multiple pterygium with arthrogryposis, which is found in 91% of Escobar patients with a variable range of gravity [11]. Consistent with these symptoms, three patients (Patients 1–3) had more severe joint stiffness than the other patients and had been operated on several times but relapsed despite physiotherapy and bracing. In addition, arthrogryposis in our patients was associated with rare orthopedic manifestations such as camptodactyly, hip dislocation, vertical talus, and reduced muscle bulk.

Four of our patients had scoliosis. Spinal deformity was severe in three patients (Patients 1–3), which required surgical correction. As for Patient 5, her scoliosis was mild, which stopped following bracing.

Interestingly, two patients (Patients 1 and 4) presented a pterygium in the groin region, which has been described only once in Escobar syndrome [37]. In addition, Patient 4 suffered from hip dislocation, which has never been described in this syndrome.

### 4.2. Molecular Diagnosis

#### 4.2.1. Genetic Investigation of genes Responsible for Escobar Syndrome

**Figure 6:**
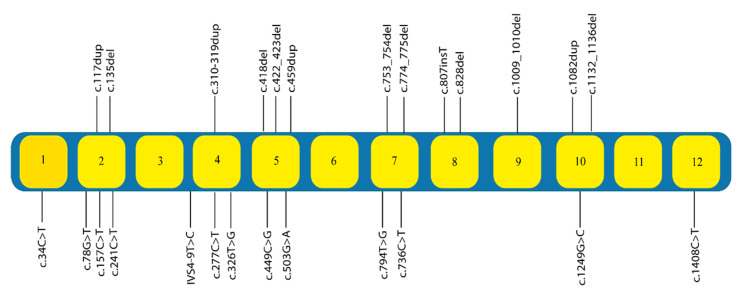
Schematic presentation of variants in *CHRNG* gene associated with Escobar syndrome (according to the literature).

The prevalence of Escobar syndrome in Tunisia and worldwide is unknown. To date, 83 patients with Escobar syndrome with mutations in *CHRNG* and *TPM2* have been reported in the literature. Generally, Escobar syndrome is associated with mutations in the *CHRNG* gene (reported in 75 Escobar patients). In detail, 26 different variants in the *CHRNG* gene were reported (Figure 6), with 2 recurrent variants located in exon 5 (c.459dup; (p.V154Sfs*24)), detected in 16 patients [11,13,20], and a deletion (c.753_754del (p.Val253Alafs*44)) in exon 7, described in 7 patients [11]. These two mutations were associated with Escobar syndrome in a compound heterozygous state that was reported previously in one Tunisian patient. It was the sole genetic study carried out in Tunisia for this pathology [15].

In our study, a homozygous *CHRNG* variant located in exon 7 c.753_754del/rs767503038 was detected in Patients 4 and 5. This is a frameshift variant that leads to a premature stop codon (p.Val253Alafs*44). It is described as pathogenic in ClinVar. This deletion is one of the most frequent mutations associated with Escobar syndrome [11]. It was described in a compound heterozygous state in a Tunisian patient with a particular phenotype, which is the absence of pterygium [15].

This variant (rs767503038) is located in the extracellular domain of the γ-subunit AChR protein, similar to the majority of mutations described in Escobar patients [11]. This domain is of great importance in acetylcholine receptor formation, as it forms a ligation site with the α-subunit.

In our study, we identified in Patient 1 a heterozygous novel splice site variant (c.351-1G>A), located in exon 5 of the *CHRNG* gene. This variant was predicted as pathogenic using multiple prediction tools, suggesting a loss of the acceptor splice site of exon 5.

Given the fact that Escobar syndrome is an autosomal recessive disorder, and that this variant (c.351-1G>A) was found in a heterozygous state in Patient 1 and her healthy mother, a second variant could be present in another region such as UTR regions or intronic regions, which could affect the expression of the gene inherited from the healthy father who we failed to identify, as we sequenced only the coding regions. In the literature, five patients were described with a heterozygous compound mutation causing Escobar syndrome [13,15]. Other studies suggested the presence of variants in the regulatory intronic regions in Escobar patients given their absence in exons and other genes associated with this pathology, such as *CHRNA1*, *CHRND, TPM2,* and *MYH3* [4,38,39]. Other studies, could not associate the phenotype with any particular related genetic variant

While *CHRNG* mutations caused Escobar syndrome for 75 patients of known cases [6], a number of patients had a genetic etiology associated with variants in the *TPM2, CHRND, CHRNA1, MUSK, MYH3, RAPSN,* and *DOK7* genes. Through whole-exome sequencing, we identified a homozygous variant in exon 6 of the *TPM2* gene NM_003289.4 (c.628C>T; p.Q210*) in two patients (Patients 2 and 3). These are the first cases reported in Tunisia harboring this variant.

The *TPM2* gene encodes the β-tropomyosin protein. It plays a crucial role in muscle contraction and stabilization of the thin filament [40].

Mutations in the *TPM2* gene are generally associated with autosomal dominant diseases, such as distal arthrogryposis (DA) [41], cap myopathy disease [42], nemaline myopathy 4 (NEM4) [43], and congenital fiber-type disproportion (CFTD) [44], and rarely associated with Escobar syndrome with autosomal recessive transmission mode.

Only three papers described mutations in the *TPM2* gene associated with Escobar syndrome [17,18,19]. The variant identified in our patients was previously reported in an Algerian consanguineous family [17]. It was described in four patients with Escobar syndrome associated with nemaline myopathy. For these Algerian patients, they developed late motor acquisition and severe scoliosis, in addition to classical clinical features of Escobar syndrome.

The patients (Patients 2 and 3) harboring the variant in the *TPM2* gene were from Northwest Tunisia (Bizerte). Patient 2 was born from an endogamous marriage, while Patient 3 was from a consanguineous family. The high rate of consanguinity and endogamy in Tunisia has led to an increase in the frequency of rare diseases. More specifically, Bizerte is considered among the governorates with a high rate of inbreeding (around 39.3%) [45]. This may be the cause of the disease emergence for both patients and the presence of other family members who did not consult.

Moreover, the recurrence of the *TPM2* variant in the same ethnic group from North Africa in Tunisian and Algerian patients [17] suggests that it could be a mutation with a founder effect. Haplotype analysis could be appropriate to prove this hypothesis.

The variant detected in our patients leads to the appearance of a premature stop codon at amino acid position 210 (p.Q210*). This leads to the degradation of the RNA by the nonsense-mediated mRNA decay (NMD) process. This was confirmed by the absence of *TPM2* gene expression [17].

NM is a rare congenital disorder that affects skeletal muscles [46]. It is phenotypically and genetically very heterogeneous. Clinically, it is characterized by hypotonia and muscle weakness at birth or in early childhood and, in some cases, adulthood. In this work, the EMG result was within the lower normal range for motor and sensory nerve conduction and low-frequency stimulations. However, the myogenic EMG trace was not conducted. This test was not performed for Patient 3, as she was not available. In fact, EMG is considered a diagnosis tool for neuromuscular disorders; however, it is not recommended for the diagnosis of NM. Most cases with NM presented a normal EMG result. This is why for NM the diagnosis is based on muscle biopsy analysis for histological examination [47]. As in our study, we could not get access to muscle biopsies to confirm histological damage.

#### 4.2.2. Investigation of the Clinical Particularities in Patient 3

Mutations in the *TPM2* gene could explain some symptoms of Escobar syndrome such as distal arthrogryposis, psychomotor delay, and reduced muscle bulk observed in distal arthrogryposis [48].

In addition to the typical phenotype of Escobar syndrome, Patient 3 displayed lower limb paraplegia, which was not observed in Patient 2 with the same *TPM2* genotype. To our knowledge, none of the *TPM2* mutations or even NM are associated with paraplegia. Therefore, the presence of the c.628C>T; p.Q210* variant in the *TPM2* gene could not explain this particular clinical feature. These phenotypic outcomes could further support the existence of genetic modifiers in Patient 3. We hence performed a deeper genetic investigation to explain the variability between both patients (Patients 2 and 3) using WES data. Indeed, we identified three novel variants predicted as pathogenic using different bioinformatics tools, which were specific to Patient 3. The presence of additional rare predicted-deleterious variants and phenotypic severity support the existence of genetic modifiers or oligogenic variants. Two of these variants are located in kelch-like genes. In recent decades, several studies have shown the implication of different kelch-like proteins in skeletal muscle development that coordinate proliferation, cell migration, and differentiation of the muscle. As a result, the dysregulation of these proteins is implicated in skeletal muscle diseases [49].

In our study, we reported a novel heterozygous sporadic variant in the *KLHL30* gene. *KLHL30* is a hub gene that controls the development process of several network pathways [50]. It is one of the skeletal muscle genes that were upregulated during both phenomena of regrowth and hypertrophy of muscle in a mouse model [51]. Few reports have studied the role of this gene in humans, where they suggest its implication in osteogenic differentiation through the control of fatty acid metabolism [52]. We therefore hypothesize that the presence of the c.632G>A variant in Patient 3 may be behind the severe bone deformities and muscle weakness and contribute to her paralysis. The second variant in the kelch-like gene family was located in the *KLHL40* gene, inherited from her healthy father. Kelch-like protein 40 (KLHL40) or KBTBD5 is a myostructural protein of fetal skeletal muscle. It is also expressed during adulthood and plays a role in myoblast differentiation [53,54] and interacts with E3 ubiquitin ligases [49]. Mutations in the *KLHL40* gene were related to severe nemaline myopathy type 8 (NEM8; MIM 615348) with recessive inheritance, including clinical signs of akinesia, arthrogryposis, hypotonia, chest deformities, respiratory problems, and fractures, leading to death in early childhood [47,53]. In fact, some of these clinical features were present in Patient 3 (hypotonia, chest deformities, and progressive arthrogryposis), which could be associated with this variant. Furthermore, a previous report suggested that alterations of *KLHL40* lead to impaired muscle contractility, sarcomeric disorganization [55], and an increase in the expression of actin [53], which is likely to be associated with muscle weakness. This could explain the paraplegia and akinesia in Patient 3 as well.

The third heterozygous variant in the *CACNA1S* gene was inherited from her healthy mother. Calcium channel alpha 1a subunit (*CACNA1S*) is the major subunit (pore-forming) of the dihydropyridine receptor (DHPR). This receptor plays an important role in the process of the excitation–contraction coupling of skeletal muscles. It is located in the outer membrane of tubule T of the membrane muscle that transmits electrical signals (PA) to activate another calcium receptor called ryanodine receptor 1 (RYR1), which leads to the release of calcium from sarcoplasmic reticulum (SR) storage. Mutations in *CACNA1S* are mostly related to dominant inheritance, causing hypokalemic periodic paraplegia type 1 (MIM 170400) and malignant hyperthermia (MIM601887). Recently, variants in this gene were related to congenital myopathy with autosomal recessive and dominant inheritance [56].

These four altered genes (*TPM2, KLHL30, KLHL40,* and *CACNA1S*), detected using WES, are expressed in the muscle and have been associated in the literature with akinesia, NM, and arthrogryposis. In fact, the *TPM2* variant explains the Escobar phenotype but does not explain the paraplegia in Patient 3. Muscle innervation reaches the T-tubules of the myotubes and activates the voltage-gated L-type Ca^2+^ channel, which includes CACNA1S as a major subunit. This pathway subsequently stimulates the troponin–tropomyosin (β-tpm) and actin complex, leading to muscle contraction. This complex is stabilized by KLHL40 proteins, allowing normal sarcomere function [55]. Therefore, any alteration of this process generally leads to myopathy.

These variants could in part explain the myopathy; nevertheless, further investigations are required to explain the paraplegia observed in Patient 3, which could be related to neurological manifestations.

### 4.3. Cases of LMPS in Our Cohort

Another particularity in our study is that both families with *TPM2* variation described a history of multiple spontaneous abortions; they harbored an intronic variant g.2975T>A of reference rs6761667 in the *CHRNG* gene. This variant was associated with high blood pressure in the German population following genetic association tests [57]. Hence, our hypothesis is that this variant could be associated with LMPS in the families of Patients 2 and 3. Furthermore, metabolic disturbance was associated in some cases with fetal akinesia [58,59], which is in concordance with our hypothesis.

As for the two patients with recurrent variants in exon 7 of *CHRNG,* a previous report suggests that the same mutation could present in a lethal or nonlethal form. Through inquiry, the sibling of Patient 4 died 6 months after birth from respiratory deficiency and severe impaired growth of skeletal and pulmonary muscles, in addition to multiple pterygium and arthrogryposis. As for the family of Patient 5, the mother has had several spontaneous abortions due to the lack of fetal development. This mutation was previously associated with Escobar syndrome and LMPS forms [6,60].

### 4.4. Investigation of Candidate Genes’ Expression Associated with Escobar Syndrome: IGF-1 and POLG1

#### 4.4.1. IGF-1 Expression

In our cohort, four out of five patients had short stature, which characterizes 55% of Escobar patients [11]. Many genes are involved in musculoskeletal development; among them is the insulin-like growth factor-1 (*IGF-1*) gene [25]. It plays a role in the development of long bones and vertebrae by stimulating the chondrogenesis process [23,25,61]. This gene is associated with idiopathic short stature in many cases [25,62]. The alteration of *IGF-1* is not only associated with short stature but is also implicated in inflammation, neurological disorders, metabolic diseases, and aging (it declines after the third decade) [63]. Therefore, in order to explain the growth retardation in our patients, we analyzed *IGF-1* expression. We found a downregulation of this gene in four patients presenting short stature, except Patient 4, who had a normal height.

In addition, *IGF-1* is expressed during the development of the fetus. It is crucial for intrauterine growth and postnatal skeletal growth [64,65]. The low expression of this gene could be the cause of fetal akinesia, reduced muscle bulk, and bone deformities in Escobar patients.

#### 4.4.2. POLG1 Expression

Gomori trichrome staining is used in typical cases of mitochondrial myopathy and in nemaline myopathy, to show an accumulation of bloated-up mitochondria called megaconia at the periphery of muscle fibers [66] and to detect the nemaline rods. This was previously observed in Escobar patients harboring *TPM2* mutations [17]. Since DNA polymerase gamma 1 is a protein essential for mitochondrial replication and stability [29], we sought to explore possible interaction between *TPM2* and *POLG1*. Unfortunately, no muscle biopsies were available for such explorations. We therefore explored *POLG1* expression in Escobar patients with or without *TPM2* variations. We found overexpression of *POLG1* in Patients 2 and 3 but normal *POLG1* expression in the other patients with *CHRNG* mutation. This could explain the reduced muscle bulk and muscle weakness in our Escobar patients with TPM2 defects and confirm the NM observed in similar phenotypes in a neighboring community of Algerian Escobar patients [27].

## 5. Conclusions

In conclusion, we reported the largest cohort of Escobar patients in North Africa for whom we identified a mutational spectrum. We identified variants in the *TPM2* and *CHRNG* genes that highlight the genetic heterogeneity of this syndrome. These results will be useful for confirming clinical diagnosis and setting up prenatal diagnosis, and genetic counseling for families at risk.

Furthermore, our work highlights clinical heterogeneity that may be due to other genetic modifier factors. In addition, we report, for the first time, two genes whose expression analysis provides information on the severity of certain clinical features, such as growth retardation (for *IGF-1*), and helps to improve clinical diagnosis by distinguishing between two forms of Escobar syndrome with or without nemaline myopathy (for *POLG1*).

These results implicate pathways that have not been previously investigated in Escobar syndrome and which should be explored in depth to better understand the physiopathology of this disease.

## Figures and Tables

**Table 1:** Primers of qPCR.

Primer Name	Primer Sequence	Temperature (°C)
*IGF-1*-Forward	GCTGGTGGATGCTCTTCAGT	60
*IGF-1*-Reverse	ACCCTGTGGGCTTGTTGAAA	62
*POLG1*-Forward	GAGAAGGCCCAGCAGATGTA	60
*POLG1*-Reverse	ATCCGACAGCCGATACCA	60
*PPIA*-Forward	TTTCATCTGCACTGCCAAGA	60
*PPIA*-Reverse	TTGCCAAACACCACATGCT	61
*RPLP0*-Forward	TGCATCAGTACCCCATTCTATCA	61
*RPLP0*-Reverse	AAGGTGTAATCCGTCTCCACAGA	62

**Table 2:** Clinical and radiological features of our patients with Escobar syndrome.

	Patients				
Clinical Signs	Patient 1	Patient 2	Patient 3	Patient 4	Patient 5
Ethnicity	Tunisia: Bizerte	Tunisia: Bizerte	Tunisia: Bizerte	Tunisia: Kebeli	Tunisia: Tataouin
Gender (Male/Female)	Female	Female	Female	Female	Female
Age (Years)	10	15	23	9	9
Number of Cases Within the Same Family	1	2	1	1	1
Consanguinity	Endogamy	Endogamy	Consanguineous	Consanguineous	Consanguineous
Molecular Diagnosis	*HTZ CHRNG* gene (NM_005199.5) exon 5 c.351-1G > A/ rs: 761413806	*HMZ TPM2* gene (NM_003289.4); exon 6 c.628C > T; p.Q210*/rs199476154	*HMZ TPM2* gene (NM_003289.4); exon 6 c.628C > T; p.Q210*/rs199476154	*HMZ CHRNG* gene (NM_005199.5) exon 7 c.753_754del; p.Val253Alafs*44/rs767503038	*HMZ CHRNG* gene (NM_005199.5) exon 7 c.753_754del; p.Val253Alafs*44/rs767503038
History of Spontaneous Abortion/LMPS	−	+	+	+	+
Short Stature	+	+	+	−	+
Height (m)	1.35	1.40	1.35	1.30	1.20
Weight (kg)	50	46	45	26	12
Reduced Fetal Movement	NI	NI	+	+	NI
Facial Dysmorphism	Ptosis, small mouth, low nose bridge	Ptosis, small mouth, low nose bridge	Ptosis, small mouth, low nose bridge	Ptosis, small mouth, low nose bridge	Ptosis, small mouth
Short Neck	+	+	+	+	+
Low-Set Ears	+	+	+	+	+
Motor Development	Delayed	Delayed	Delayed	Delayed	Delayed
Walking Impairment	−	+(with assistance)	+(wheelchair)	−	−
Arthrogryposis	+	+	+	+	+
Multiple Pterygium	+	+	+	+	+
Neck Pterygium	+	+	+	+	+
Axilla Pterygium	+	+	+	+	+
Elbows Pterygium	+	+	+	+	+
Knees Pterygium	+	+	+	+	+
Camptodactyly	+	+	+	+	+
Bilateral Vertical Talus	+	+	+	+	+
Respiratory Involvement	−	−	+	−	−
Scoliosis	Lumbar scoliosis	Severe dorsal scoliosis	Lordoscoliosis	−	Lordoscoliosis
Scoliosis: Cob Angle before Treatment	60°	80°	−50°	−	25°
Orthopedic treatment/Physiotherapy	Bracing physiotherapy	Bracing Physiotherapy and knee splinting	Physiotherapy and knee splinting Halo-gravity traction	Orthopedic shoes	
Surgical Treatment For:	+	+	+	+	+
Flexion Contracture of the Knees	Ilizarov external fixator Distal femur osteotomy	Bilateral soft-tissue release Distal femur osteotomy	Bilateral soft-tissue release	Distal femur osteotomy	Distal femur osteotomy
Bilateral Vertical Talus	+	+	+	+	
Hip Dislocation	Soft-tissue release	−	−	+ pelvic osteotomy	
Surgical Intervention for the Correction of the Scoliosis	Dual traditional growing rods and pelvic fixation Spinal arthrodesis	Single traditional growing rods	Anterior and posterior arthrodesis	−	
Cob Angle of the Scoliosis after Surgical	40°	40°	−20°	−	−
Conclusion of the EMG	Lower acceptable range for the nerve conductance and velocity	Lower acceptable range for the nerve conductance and velocity	NI	Lower acceptable range for the nerve conductance and velocity	NI
Other	- Pelvic obliquity - Pterygium in the groin and inguinal region	- Reduced muscle bulk and fatigability in lower limbs	- Impaired sitting ability - Reduced muscle bulk and fatigability in lower limbs	- Bilateral hip dislocation - Pterygium in the groin and inguinal region	−

NI: not identified. HTZ: heterozygous. HMZ: homozygous. +/− presence or absence

**Table 3:** Prediction of the pathogenicity of the splice site mutation.

Prediction Site	Score	Significance
Mutation Taster	1	Pathogenic
Human Splicing Finder	55.45	Alteration of the site
Maximum entropy model	−2.58	Not predicted as a splice site
Markov Model	−2.61	Not predicted as a splice site
Weight Matrix Model	−3.83	Not predicted as a splice site
VarSEAK	−3.27% (class5 splicing affect)	exon skipping, loss of function

The investigation of the second variant is still in progress using WGS technology.

**Table 4:** Disease-causing digenic combinations scores of oligogenic interaction of the different genes found in Patient 3 using Orval tool.

Variation in the Different Gene Pairs	Classification Score	Support Score	Predicted Class	Confidence Zone
***KLHL40*** ***CACNA1S***	0.8671	100.00	Disease causing	99% confidence
***KLHL30*** ***CACNA1S***	0.7400	99.20	Disease causing	>95% confidence
***TPM2*** ***CACNA1S***	0.5500	55.00	Disease causing	90%

## Data Availability

All processed data have been provided in the manuscript. Raw data, generated for this study, can be provided by the corresponding author upon reasonable request.
